# Gene Acquisition by a Distinct Phyletic Group within *Streptococcus pneumoniae* Promotes Adhesion to the Ocular Epithelium

**DOI:** 10.1128/mSphere.00213-17

**Published:** 2017-10-25

**Authors:** Irena Antic, Kimberly M. Brothers, Maureen Stolzer, Han Lai, Evan Powell, Rory Eutsey, Rolando A. Cuevas, Xinyu Miao, Regis P. Kowalski, Robert M. Q. Shanks, Dannie Durand, N. Luisa Hiller

**Affiliations:** a Department of Biological Sciences, Carnegie Mellon University, Pittsburgh, Pennsylvania, USA; b Department of Ophthalmology, University of Pittsburgh, Pittsburgh, Pennsylvania, USA; c Center of Excellence in Biofilm Research, Allegheny Health Network, Pittsburgh, Pennsylvania, USA; Centers for Disease Control and Prevention

**Keywords:** *Streptococcus pneumoniae*, gene transfer, genomics, host-pathogen interactions, phylogenetic analysis

## Abstract

*Streptococcus pneumoniae* (pneumococcus) displays broad tissue tropism and infects multiple body sites in the human host. However, infections of the conjunctiva are limited to strains within a distinct phyletic group with multilocus sequence types ST448, ST344, ST1186, ST1270, and ST2315. In this study, we sequenced the genomes of six pneumococcal strains isolated from eye infections. The conjunctivitis isolates are grouped in a distinct phyletic group together with a subset of nasopharyngeal isolates. The keratitis (infection of the cornea) and endophthalmitis (infection of the vitreous body) isolates are grouped with the remainder of pneumococcal strains. Phenotypic characterization is consistent with morphological differences associated with the distinct phyletic group. Specifically, isolates from the distinct phyletic group form aggregates in planktonic cultures and chain-like structures in biofilms grown on abiotic surfaces. To begin to investigate the association between genotype and epidemiology, we focused on a predicted surface-exposed adhesin (SspB) encoded exclusively by this distinct phyletic group. Phylogenetic analysis of the gene encoding SspB in the context of a streptococcal species tree suggests that* sspB* was acquired by lateral gene transfer from *Streptococcus suis*. Furthermore, an *sspB* deletion mutant displays decreased adherence to cultured cells from the ocular epithelium compared to the isogenic wild-type and complemented strains. Together these findings suggest that acquisition of genes from outside the species has contributed to pneumococcal tissue tropism by enhancing the ability of a subset of strains to infect the ocular epithelium causing conjunctivitis.

**IMPORTANCE** Changes in the gene content of pathogens can modify their ability to colonize and/or survive in different body sites in the human host. In this study, we investigate a gene acquisition event and its role in the pathogenesis of *Streptococccus pneumoniae* (pneumococcus). Our findings suggest that the gene encoding the predicted surface protein SspB has been transferred from *Streptococcus suis* (a distantly related streptococcal species) into a distinct set of pneumococcal strains. This group of strains distinguishes itself from the remainder of pneumococcal strains by extensive differences in genomic composition and by the ability to cause conjunctivitis. We find that the presence of *sspB* increases adherence of pneumococcus to the ocular epithelium. Thus, our data support the hypothesis that a subset of pneumococcal strains has gained genes from neighboring species that enhance their ability to colonize the epithelium of the eye, thus expanding into a new niche.

## Abstract (precis)

Changes in the gene content of pathogens can modify their ability to colonize and/or survive in different body sites in the human host. In this study, we investigate a gene acquisition event and its role in the pathogenesis of *Streptococccus pneumoniae* (pneumococcus). Our findings suggest that the gene encoding the predicted surface protein SspB has been transferred from *Streptococcus suis* (a distantly related streptococcal species) into a distinct set of pneumococcal strains. This group of strains distinguishes itself from the remainder of pneumococcal strains by extensive differences in genomic composition and by the ability to cause conjunctivitis. We find that the presence of *sspB* increases adherence of pneumococcus to the ocular epithelium. Thus, our data support the hypothesis that a subset of pneumococcal strains has gained genes from neighboring species that enhance their ability to colonize the epithelium of the eye, thus expanding into a new niche.

## INTRODUCTION

Streptococcal species are characterized by extensive intraspecies gene diversity that plays critical roles in tissue tropism and, consequently, disease outcomes (1–3). In group A streptococci (GAS), a significant association has been detected between gene content and the ability to colonize the skin versus the upper respiratory tract, leading to either impetigo or pharyngitis (1). In contrast, in the human pathogen *Streptococcus pneumoniae* (pneumococcus), an association between genomic background and site of infection had not been evident (4). The absence of this association is unexpected given the large number of diverse pneumococcal lineages and the variety of body sites that can be infected by this bacterium (2, 5, 6). Pneumococcus colonizes the nasopharynx, from where it can disseminate to tissues: frequently the middle ears and lungs and relatively rarely the eyes, heart, and brain (7). Pneumococcal conjunctivitis is a notable exception to the lack of association between phylogenic background and site of infection: the genomic composition of strains causing pneumococcal conjunctivitis differs from that of strains causing disease in other tissues. This difference in gene content is so extensive that this set of strains localizes to a distinct phyletic group (8–10).

The best-characterized feature shared across conjunctivitis-associated pneumococcal strains is the absence of the genes encoding the polysaccharide capsule (8–11). The nonencapsulated strains in the distinct phyletic group are referred to as the classical nonencapsulated strains (8, 11). In the region syntenic to the capsular locus, these strains encode *aliC* and *aliD*, which are putative lipoproteins of unknown functions (9, 12–14). The capsule is the main pneumococcal virulence determinant: thus, the absence of the capsule has important clinical implications for the distinct phyletic group. Nonencapsulated strains are less virulent than encapsulated strains and are much less likely to disseminate in single-strain infections (15). Furthermore, the capsular structure is the target of the pneumococcal vaccine: thus nonencapsulated strains escape the vaccine (11).

Whereas the absence of capsule is a shared feature of all strains in the distinct phyletic group, this feature alone cannot be used as a marker. Many nonencapsulated strains are phylogenetically clustered with the majority of pneumococcal strains (16, 17). Nonencapsulated strains organized into the major pneumococcal phylogenetic branch are referred to as the sporadic nonencapsulated strains (8, 11). A subset of nonencapsulated strains has its origin in encapsulated strains that no longer encode the capsule due to mutations or deletions in the capsular locus; these are termed group I (11–13). The remainder of sporadic nonencapsulated strains, group II, carry noncapsular genes syntenic to the capsular ones (11). This locus carries either *pspK* (also known as *nspA*) or *aliD* (also known as *aliB* open reading frame 2 [ORF2]) (13). The strains carrying *aliD* may also carry *aliC* (also known as *aliB* ORF1) (11–14). Thus, the absence of capsule is a highly clinically relevant feature shared by conjunctivitis strains but not what drives the association between genomic background and ability to cause conjunctivitis.

Pneumococcal eye disease is not limited to conjunctivitis, where it infects the conjunctival epithelial layers. This pathogen can also infect the vitreous body inside the eye (endophthalmitis) and the cornea (keratitis) (18–20). It is unclear whether there are morphological and genomic features shared by all isolates that infect the human eye. In this study, we combine genomics, phylogenetics, and cell adhesion studies to gain insight into the genomes of isolates from multiple types of pneumococcal ocular infections and the tissue tropism associated with conjunctivitis isolates.

## RESULTS

### Sequencing of pneumococcal strains isolated from ocular infections.

To compare the genomes of pneumococcal strains isolated from multiple types of eye infections, we sequenced six eye-associated strains. One isolate is from an endophthalmitis infection (strain E709), three are from keratitis infections (strains K2521, K2527, and K2557), and two are from conjunctivitis infections (strains B1598 and B1599). All isolates are deidentified clinical samples obtained at the Charles T. Campbell Ophthalmic Laboratory at the University of Pittsburgh Eye Center from October 2012 to May 2013. The strains were sequenced using single-molecule real-time (SMRT) technology, and the genome sequences have been deposited in GenBank (Table 1).

**TABLE 1:** Description of strain isolation, sequencing and assembly, and drug resistance profiles for eye-associated *S. pneumoniae* strains

Parameter	Result for strain:					
	E709	K2521	K2527	K2557	B1598	B1599
Isolation date (mo/day/yr)	5/13/13	1/28/13	2/21/13	6/19/13	10/19/12	11/3/12
Isolation site	UPMC	UPMC	UPMC	UPMC	UPMC	UPMC
Serotype^a^	23A	15B	19A	17F	NE	NE
MLST	338	3280	320	2355	2315	2315
Accession no.						
GenBank	JBOR00000000	JBOS00000000	JBOT00000000	JBOU00000000	JBOV00000000	JBOW00000000
BioProject	PRJNA235160	PRJNA235267	PRJNA235268	PRJNA235269	PRJNA235270	PRJNA235271
BioSample	SAMN02691894	SAMN02691895	SAMN02691896	SAMN02691897	SAMN02691898	SAMN02691899
CG (%)	39.6	39.6	39.8	39.8	39.6	39.6
No. of contigs	37	32	29	111	158	38
Read coverage (×)	55.22	73.71	63.13	30.39	52.07	49.71
Sequencing platform	SMRT	SMRT	SMRT	SMRT	SMRT	SMRT
Estimated genome size (bp)	2,155,121	2,184,812	2,180,290	2,051,681	2,902,565	2,422,872
Resistance	Gentamicin, amikacin	Tobramycin, polymyxin B	Gentamicin, tobramycin, polymyxin B	Gentamicin, polymyxin B, tobramycin, sulfonamide, ciprofloxacin,^b^ ofloxacin^b^	Gentamicin, trimethoprim, polymyxin B, tobramycin	Gentamicin, trimethoprim, polymyxin B, tobramycin

a Serotypes were determined based on genome sequence. NE, nonencapsulated.

b Intermediate resistance.

Pneumococcal genomes are conventionally classified using multilocus sequence typing (MLST) (which serves as a proxy for lineages), polysaccharide capsular types, and drug resistance profiles (2, 21). The six strains encompass five STs and four capsular types, as well as nonencapsulated types, and display various levels of resistance to a standard panel of antibiotics (Table 1). The conjunctivitis strains are nonencapsulated, while the keratitis and endophthalmitis strains are encapsulated. All strains displayed some degree of drug resistance: the broadest was observed for keratitis strain K2557, which is resistant to gentamicin, polymyxin B, tobramycin, and sulfonamide and has intermediate resistance to the fluoroquinolone antibiotics ciprofloxacin and ofloxacin (Table 1). In summary, strains isolated from the different ocular infections display diverse ST, serotype, and resistance profiles.

### Phylogenetics and comparative genomics of pneumococcal strains isolated from ocular infections.

**FIG 1:**
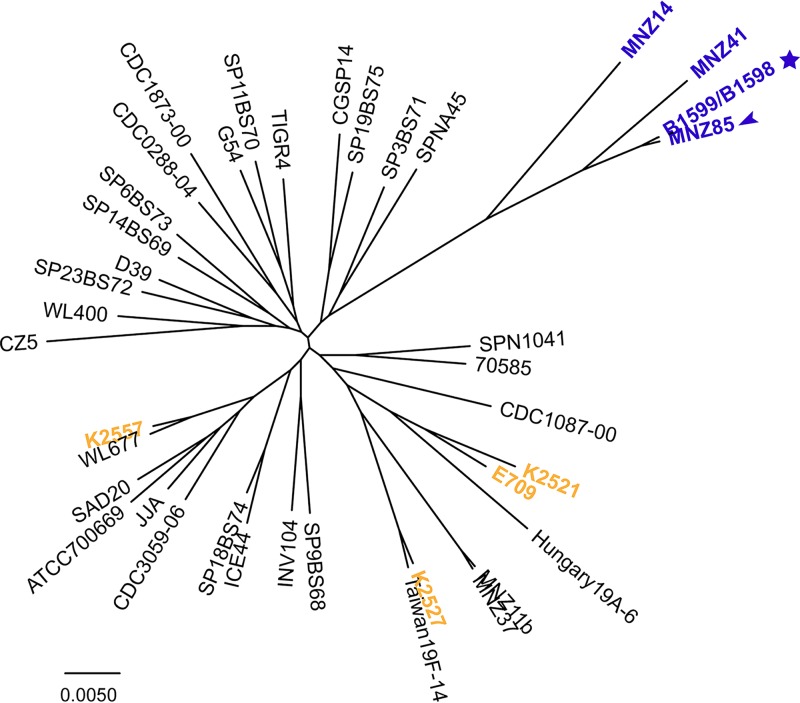
Phylogenetic analysis of pneumococcal strains. Maximum likelihood phylogeny of 40 pneumococcal strains generated from the core genome. Purple, distinct phyletic group; arrow, nasopharyngeal isolate; star, conjunctivitis isolates; yellow, endophthalmitis- and keratitis-associated strains. The scale bar indicates the number of substitutions per site. The numbers on the branch denote bootstrap values. The basal position of the distinct phyletic group presented in the context of other streptococcal species is illustrated in Fig. S3.

To establish the phylogenetic relationship of these eye-associated strains, we compared the six genomes with a diverse set of 34 pneumococcal genomes from strains isolated from blood, lung, and nasopharynx (see Table S1 in Data Set S2 in the supplemental material). This highly curated set consists of genomes used for the first large-scale pneumococcal pangenome studies (2, 5), genomes from PCV-7-immunized children (22), and genomes from nonencapsulated isolates (23). Together this set reflects diversity in multilocus sequence types, serotypes, disease states, and geographical locations. We generated and aligned the core genomes of these sequences and produced a maximum likelihood phylogenetic tree (Fig. 1). In agreement with published work (8, 9, 24), the most prominent feature of the phylogenetic tree is the presence of a distinct and strongly supported branch that contains the conjunctivitis strains (Fig. 1).

A second noteworthy feature of the phylogenetic tree is the tight grouping between conjunctivitis and nasopharyngeal strains in the distinct phyletic group. Specifically, there is a short distance between the conjunctivitis strains B1599 and B1598 and the nonencapsulated nasopharyngeal carriage strain MNZ85; furthermore, there is strong support for the branch that groups these strains together (Fig. 1). The mixture of nasopharyngeal and conjunctivitis isolates on this distinct phyletic group suggests that diversification of these strains is not a consequence of niche isolation and instead supports a model where strains migrate between the ocular epithelium and the nasopharynx. Finally, in stark contrast to the conjunctivitis strains, the endophthalmitis and keratitis isolates are not part of the distinct phyletic group (Fig. 1, yellow). The three keratitis isolates are distributed across the main group, suggesting the core genome is not associated with the ability of strains to cause keratitis. This arrangement demonstrates that belonging to the distinct phyletic group is not a feature shared by all strains that infect the eye—rather, it is a distinguishing feature of conjunctivitis. In summary, phylogenetic analysis of six pneumococcal strains isolated for three types of eye infection revealed that conjunctivitis isolates comprise a distinct phyletic group together with nonencapsulated nasopharyngeal strains, while keratitis and endophthalmitis isolates are clustered with the remainder of strains.

To identify the genes that distinguish the distinct phyletic group from other pneumococcal strains, we singled out the coding sequences (CDSs) that are present in the conjunctivitis isolates (B1598 and B1599) but absent in strains from the other major branch. We identified 77 coding sequences, many of which were grouped together in the genome (see Fig. S1 in the supplemental material). The most widespread functional feature of these genes is a predicted localization to the bacterial surface: that is, surface localization motifs (LPXTG or YSIRK), surface-related functions (such as ABC transporter), and/or motifs predicted to interact with the host (β-galactosidase, choline binding, sialidase, and two adhesins) (10, 25–29).

10.1128/mSphere.00213-17.1 FIG S1  Genes enriched in the distinct phylogenetic branch. (Left column) List of 77 genes present in conjunctivitis isolates (B1598 and B1599) but absent in strains from the other major branch, including those isolates from keratitis and endophthalmitis. The numbers represent the locus number from genome B1599 (GenBank accession no. JBOW00000000). Colors denote genes grouped on the chromosome, and asterisks denote genes predicted to be surface exposed. The heat map summarizes the BLASTp analysis of the coding sequences this study identified as unique to the distinct phylogenetic branch relative to their distribution in a large set of isolates gathered by Croucher and colleagues (6). The comparative set consists of 616 strains: 10 from the SC12 cluster that corresponds to the distinct phyletic branch, and 606 from 15 monophyletic sequence clusters and one additional diverse group of less common genotypes (6). Of the 616 genomes, only those with one or more hits are presented in the heat map. Yellow, positive hit; blue, no hit. The hits in SC12 can be organized into two distributions: (i) genes rare in the SC12 group (30% or less of the genomes) and (ii) genes present in 90 to 100% of the SC12 genomes. All of the genes are rare outside the SC12 group and are candidates for biological features that distinguish the distinct phyletic group from other pneumococcal strains. Predicted CDSs are provided in Data Set S1 in the supplemental material. Download FIG S1, TIF file, 2.6 MB. Copyright © 2017 Antic et al. 2017 Antic et al. This content is distributed under the terms of the Creative Commons Attribution 4.0 International license.

10.1128/mSphere.00213-17.6 DATA SET S1  Multi-FASTA file of the predicted coding sequences presented in Fig. S1. Download DATA SET S1, TXT file, 0.1 MB. Copyright © 2017 Antic et al. 2017 Antic et al. This content is distributed under the terms of the Creative Commons Attribution 4.0 International license.

The association between this distinct phyletic group and conjunctivitis isolates (prominently those of ST448 and ST344) has been observed in three independent large-scale genomic studies (8–10). To compare our finding to previous work, we utilized a set of 616 genomes isolated in Massachusetts from 2007 to 2010 (6, 24). This set includes 10 strains from the distinct phyletic group identified by Croucher and colleagues (24), termed SC12, as well as 606 additional genomes. The data sets are highly consistent: genes captured exclusively in our distinct phyletic group are present in multiple SC12 genomes and are either absent or rare (<2%) in the 606 non-SC12 genomes (Fig. S1). The set of uncharacterized CDSs, predicted to be surface exposed, are likely candidates for molecular components of tissue tropism to the ocular epithelium during conjunctivitis.

### Morphological features that distinguish strains isolated from conjunctivitis relative to other eye infections.

The core (Fig. 1) and distributed (Fig. S1) genes of conjunctivitis strains and of a subset of nasopharyngeal strains differ from those of the majority of pneumococcal strains. This raises the question as to whether and how these strains differ regarding morphology.

**FIG 2:**
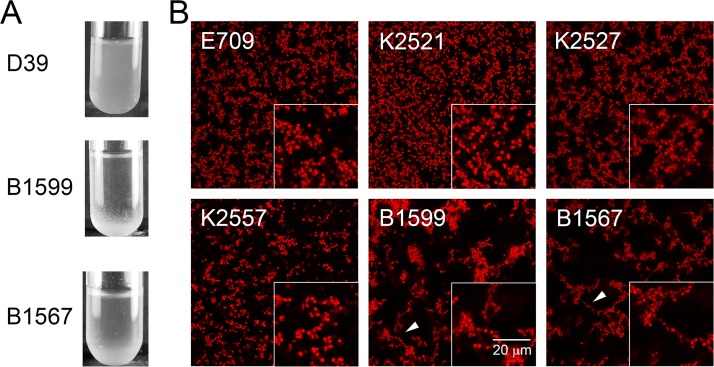
Conjunctivitis-associated strains B1599 and B1567 exhibit aggregates in planktonic culture and form abundant chain-like structures in biofilms. (A) Planktonic cultures of strains B1599, B1567, and D39. Conjunctivitis isolates precipitate at the bottom of the test tube, but no precipitate is observed for model strain D39. (B) Confocal images of 72-h biofilms fixed and stained with Syto59. Boxes on the bottom right display a magnified view. Conjunctivitis isolates B1599 and B1567 form chain-like structures (white arrows point to examples of chain-like structures); however, equivalent chains are not observed in the other eye-associated strains. The scale bar is the same for all micrographs.

To explore this question, we investigated the morphology of conjunctivitis strains in planktonic and biofilm modes of growth. The conjunctivitis strain B1599 is of multilocus sequence type (MLST) 2315. However, ST488 (represented in Fig. 1 by isolate MNZ14) is the MLST most commonly associated with conjunctivitis; thus we also analyzed strain B1567 (ST448). Strain B1567 was isolated from a patient with conjunctivitis and was selected as a representative of ST488. First, we analyzed planktonic cultures of these two conjunctivitis strains (BS1599 [ST2315] and BS1567 [ST488]), comparing them to a model strain, D39. In contrast to canonical strains, such as the control, both conjunctivitis isolates formed aggregates in stationary-phase planktonic cultures, easily visualized at the bottom of the culture tubes (Fig. 2A). Second, we investigated biofilm-related phenotypes, given that pneumococci can colonize the epithelium by growing in a biofilm mode of growth (30–35). We employed confocal microscopy to compare biofilm growth of 3-day biofilms of conjunctivitis strains with those of other ocular strains. In contrast to previously characterized pneumococcus biofilms (36) and to the keratitis and endophthalmitis isolates, the majority of cells in strain B1599 were organized into long chain-like structures, often with over 20 connected cells (Fig. 2B). The long structures were also formed in strain B1567. To our knowledge, this phenotype has not been previously reported in wild-type pneumococcal strains—only pneumococcal mutants (37, 38). These observations raise the hypothesis that strains in the phyletic group that contains the conjunctivitis isolates display phenotypic differences apparent in planktonic and biofilm growth that distinguish them from most characterized pneumococcal isolates, as well isolates from other ocular infections.

### Functional studies of pneumococcal SspB.

**FIG 3:**
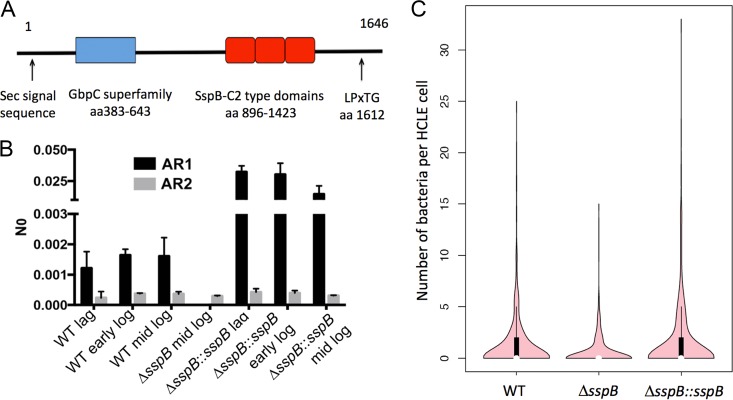
Functional analyses of the *sspB* gene encoding the predicted SspB adhesin. (A) Schematic of the predicted SspB protein (GenBank accession no. KGI30072 and OYL08640.1) illustrating domains implicated in adhesion and biofilm formation. (B) Gene expression of *sspB* in *S. pneumoniae* B1599 (black bars); for comparison, we show the expression of a second related protein with SspC-C2 domains (WP_050568636) (gray bars). The *y* axis displays *N*0, the number of fluorescence units representing the RNA amount in the respective samples. (C) Role of *sspB* in attachment to HCLE cells. HCLE cells were exposed to *S. pneumoniae* strain B1599, B1599 Δ*sspB*, or B1599 Δ*sspB*::*sspB* for 30 min, and HCLE cells with bacteria attached were enumerated. Experiments were performed in triplicate, and a total of 460 HCLE cells were analyzed for presence of bacteria and the number of bacteria attached. The results are plotted using a violin plot, generated in the R statistical package. The violin plot displays the distribution of the data: the pink areas display the density plot, the thick black bars represent the midspread of the data (interquartile range), the thin black lines display the 95% confidence interval, and the white circles correspond to the median.

To investigate the relationship between genotype and function in the distinct phyletic group, we focused on a predicted agglutinin receptor encoded exclusively by the distinct phyletic group (Fig. S1) (GenBank accession no. KGI30072 and OYL08640.1). This predicted protein contains an N-terminal Sec-type signal sequence and a C-terminal LPXTG motif, strongly suggesting it is attached to the peptidoglycan cell wall by a sortase (25, 28). It contains a glucan-binding protein C (GbpC) domain (Conserved Domain Database [CDD] E value of 8.2e−80), followed by three adhesin isopeptide-forming domains (SspB-C2 type) (CDD E values of 3.12e−69, 4.61e−68, and 3.57e−63, respectively) (39) (Fig. 3A). Proteins containing the GbpC domain are found in several species of oral streptococci, where they participate in dextran binding and biofilm formation (40, 41). SspB-C2-type domains are present in oral streptococci as components of SspB (42). In *Streptococcus gordonii*, SspB is a three-domain adhesion unit that facilitates cross-species interactions in the oral cavity (43, 44). This predicted pneumococcal sequence is 37% identical to the streptococcal surface protein B (SspB) precursor from *S. gordonii* (GenBank accession no. AAC44102.3) (45), which led to the designation of SspBC1 for the pneumococcal homolog (X231_1085) (9). To avoid confusion between the protein name and domain names, we refer to the pneumococcal protein KGI30072 as SspB. We found that the gene encoding SspB is expressed in planktonic cultures of strain B1599, as determined by quantitative reverse transcription-PCR (qRT-PCR) (Fig. 3B, black bars). The predicted surface localization and the presence of adhesive domains are consistent with a role for SspB in host interactions, and its restricted genomic distribution is consistent with a tropism to the ocular epithelium.

To test whether *sspB* has a role in adhesion to the ocular epithelium, we generated a deletion mutant (B1599 Δ*sspB*) and an overexpressor strain (B1599 Δ*sspB*::*sspB*) (see Table S2 in Data Set S2). The levels of *sspB* in the overexpression strain vary from 9- to 26-fold higher than the levels of the wild-type strains, depending on the stage of planktonic growth (Fig. 3B). The mutant and wild-type strains displayed the same morphology regarding aggregation in planktonic culture and chain-like structures in a biofilm (see Fig. S2 in the supplemental material). Thus, *sspB* alone is not responsible for the differences characterized in Fig. 2. Next, we investigated whether the gene encoding SspB influences adhesion to human corneal limbal epithelial (HCLE) cells. HCLE cells were selected because they are found at the interface between the conjunctiva and the cornea, and they are representative of the ocular surface in that they produce many mucins and compounds associated with cornea and conjunctiva (46, 47). Independently, each strain was allowed to adhere to HCLE cells, and the number of bacteria attached per cell was enumerated after gentle washing to remove nonadherent bacteria. Tukey’s test was used to establish the statistical significance of the differences in adherence to 460 HCLE cells among the three strains; these cells were gathered over three independent experiments (48). The attachment for the deletion mutant strain was significantly different from that of the wild-type (*P* = 0.0019) and complement (*P* = 0.000003) strains. Specifically, the deletion of *sspB* led to a decrease in the number of HCLE cells with any bacteria attached, as well as a decrease in the number of bacteria attached per HCLE cell (Fig. 3C). Overexpression of the gene encoding SspB in the deletion strain restored the wild-type phenotype. These data suggest that SspB plays a role in adhesion to the ocular epithelium.

10.1128/mSphere.00213-17.2 FIG S2  Strains B1599 and B1599 Δ*sspB* exhibit aggregates in planktonic culture and form abundant chain-like structures in biofilms. (Right side) WT B1599; (left side) B1599 Δ*sspB*; (top) confocal images of 72-h biofilms fixed and stained with Syto59. (The scale bar is the same for all micrographs.) (Bottom) Planktonic cultures displaying a precipitate at the bottom of the test tube. Experiments were performed in duplicate. Download FIG S2, TIF file, 6.5 MB. Copyright © 2017 Antic et al. 2017 Antic et al. This content is distributed under the terms of the Creative Commons Attribution 4.0 International license.

10.1128/mSphere.00213-17.7 DATA SET S2  Supplemental tables. Table S1 shows the pneumococcal genomes used to generate the strain tree. Boldface indicates eye-associated strains sequenced in this study. NA, not known by authors; NA—disease, site of isolation unknown, but associated with disease. Table S2 shows the strains used in this study. Table S3 is a list of *sspB* homologs used to generate a gene tree for gene-tree-species tree reconciliation. Table S4 shows the primers used in this study. Table S5 shows the nonpneumococcal strains used to generate the streptococcal tree in Fig. S2. Download DATA SET S2, XLSX file, 0.1 MB. Copyright © 2017 Antic et al. 2017 Antic et al. This content is distributed under the terms of the Creative Commons Attribution 4.0 International license.

### Origin of the pneumococcal *sspB.*

A scan of the genomic region surrounding *sspB* using CONJscan-T4SSscan software (49) identified genes encoding components of type IV secretion systems and relaxases associated with integrative conjugative elements (ICEs), consistent with a foreign origin for *sspB*. The closest relatives of the region carrying *sspB* and neighboring ICE components were identified via BLASTn search, resulting in 26 genomic regions (see Table S3 in Data Set S2). Of these, five were derived from ICE sequences, further supporting the inference that *sspB* is part of a mobile genetic element. One sequence is from outside the streptococcal genus: an ICE from *Enterococcus faecium*. Two sequences are from within *S. pneumoniae*: an ICE from isolate 403790 and a nontypeable pneumococcal strain (NT_110_58) previously localized to a distinct phyletic group (8). All other matches are streptococcal sequences from species outside *S. pneumoniae*: 13 *Streptococcus suis* strains, 2 *Streptococcus anginosus* strains, 6 strains from various pyogenic species, and one each from *Streptococcus thermophilus* and *Streptococcus gallolyticus* subsp. *macedonicus* (*Streptococcus macedonicus*).

**FIG 4:**
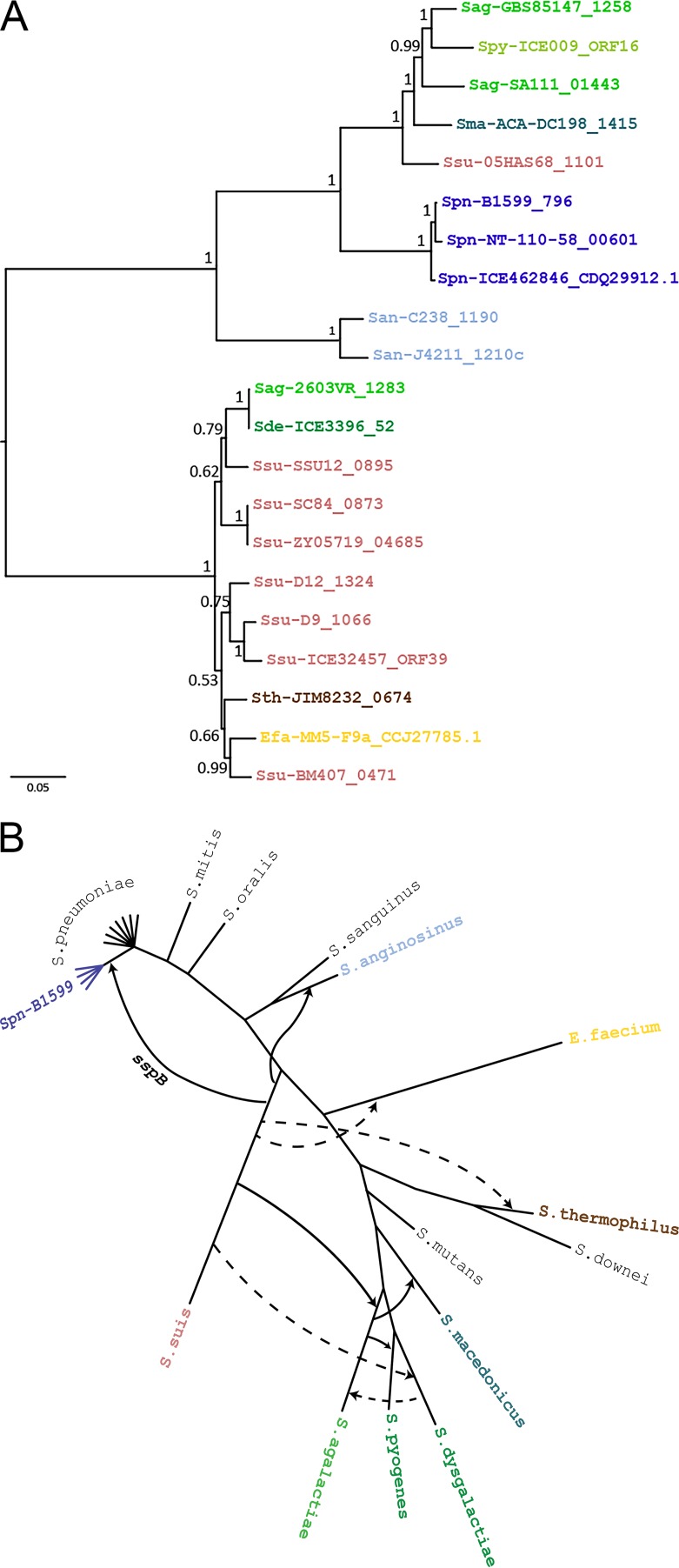
Phylogenetic reconciliation reveals a history of transfers in the origin of *sspB*. (A) Bayesian phylogeny of* sspB g*ene sequences constructed with MrBayes v.3.2.6 from codon-aware multiple alignment of nucleic acid sequences. The tree is midpoint rooted. Branches are labeled with posterior probabilities representing statistical support. The scale bar represents the number of nucleotide substitutions per site. Colors are by species according to the labeling in panel B. (B) Interspecies *sspB* gene transfers in the context of the *Streptococcus* phylogeny. Transfers were inferred by reconciling the gene tree (A) with a species tree (see Fig. S4 in the supplemental material) based on the streptococcal species tree from Richards et al. (50), generated from a core set of 136 genes sampled from 44 streptococcal species. The evolutionary history contains all species in panel A (in color), plus additional species (in black) to provide a representative sample of well-studied taxa with at least one species for each major taxonomic group. The distinct phyletic lineage in pneumococcus is shown in dark blue. Four evolutionary scenarios were inferred with Notung 2.9 (see Fig. S5 in the supplemental material), each with 9 transfers, represented as arrows from the donor to the recipient species. (Solid and dashed lines represent transfers inferred in the upper and lower subtrees in panel A, respectively.) All evolutionary scenarios support a horizontal transfer from *S. suis* to the base of the distinct lineage (dark blue).

While this patchy phyletic distribution, covering multiple dispersed groups within the genus, is suggestive of lateral gene transfer, phylogenetic analysis provides stronger evidence for lateral transfer. Explicit comparison of a gene tree with the associated species tree can distinguish between parallel loss and horizontal transfer and infer the specific transfer events that occurred. A Bayesian gene tree (Fig. 4A) was constructed from a codon-aware multiple alignment of *sspB* sequences extracted from the 21 closely related genomic regions remaining after redundant sequences, and sequences lacking ORF predictions were removed (see Table S3 in Data Set S2). Interspecies transfers were inferred by reconciling this gene tree with a previously published streptococcal species tree (see Fig. S3 in the supplemental material) (50) using Notung 2.9 (51).

10.1128/mSphere.00213-17.3 FIG S3  Phylogenetic analyses of streptococcal strains, demonstrating the basal position of the distinct phyletic group. Shown is the maximum likelihood phylogeny of multiple streptococcal species (listed in Tables S1 and S5 in Data Set S2). Colors are used to highlight species. From left to right: black, majority or pneumococcal strains; red, distinct phyletic group of pneumococcal isolates; purple, *S. pseudopneumoniae*; blue,* S. mitis*; black, *S. infantis*; green, *S. oralis* (includes strains previous annotated as *S. mitis*). The scale bar indicates the number of substitutions per site. The numbers on the branches indicate bootstrap values. The red arrow highlights the basal position of the distinct phyletic branch relative to all other pneumococcal strains. Download FIG S3, TIFF file, 0.8 MB. Copyright © 2017 Antic et al. 2017 Antic et al. This content is distributed under the terms of the Creative Commons Attribution 4.0 International license.

10.1128/mSphere.00213-17.4 FIG S4  *Streptococcus* species tree used in reconciliation. The phylogenetic reconciliation analysis was carried out with a tree representing the phylogenetic relationships of *Enterococcus faecium* and 13 *Streptoccocus* species. This tree is based on the 44-species *Streptococcus* phylogeny inferred by Richards et al. (50) from the concatenated sequences of 136 core genes. All species harboring putative *sspB* xenologs were retained in this reduced tree, as were five additional species in order to maintain a representative sample of well-studied taxa and all major taxonomic groups delineated by Richards et al. (50). For those species where an *sspB* homolog was predicted in multiple strains, all strains represented in the gene tree in Fig. 4A were added to the species tree. Strain relationships were not resolved, resulting in a terminal polytomy for each species with more than two strains. Strains are labeled with the abbreviations in Table S3 in Data Set S2. Download FIG S4, TIF file, 2.8 MB. Copyright © 2017 Antic et al. 2017 Antic et al. This content is distributed under the terms of the Creative Commons Attribution 4.0 International license.

10.1128/mSphere.00213-17.5 FIG S5  Interspecies *sspB* gene transfers in the context of the *Streptococcus* phylogeny. The* sspB* gene tree in Fig. 4A was reconciled with the species tree in Fig. S4 using Notung 2.9 to infer the event history that minimizes the weighted sum of duplications, transfers, and losses (weights of 1.5, 3.0, and 1.6). (A) Reconciliation results in four minimal evolutionary scenarios, each with 9 inferred transfers. (B to D) Evolutionary scenarios shown in the context of the species tree. All trees support the conclusion that *sspB* was first acquired by *S. suis* and later dispersed to other species though multiple gene transfer events. These scenarios differ only in the direction of gene transfers between (i) *S. dysgalactiae* and *S. agalactiae* 2603 V/R and (ii) *S. agalactiae* isolate SA111 and *S. macedonicus* strain ACA-DC 198. The fourth scenario is shown in Fig. 4B. All trees agree regarding the transfer of *sspB* from *S. suis* to *S. pneumoniae*. Download FIG S5, TIFF file, 13.9 MB. Copyright © 2017 Antic et al. 2017 Antic et al. This content is distributed under the terms of the Creative Commons Attribution 4.0 International license.

Reconciliation infers the minimal set of events that explains the topological incongruence between gene and species trees. Reconciliation with Notung 2.9 yielded four possible evolutionary scenarios with minimal event histories (Fig. 4A to D). The four scenarios are largely in agreement, differing only in the events involving pyogenic strains, which are unrelated to our central question. In particular, all four scenarios predict a single horizontal transfer from *Streptococcus suis* to *Streptococcus pneumoniae*. This suggests that the pneumococcal *sspB* was acquired from *Streptococcus suis* at the base of the distinct phyletic lineage.

## DISCUSSION

The clustering of conjunctivitis isolates into a distinct phyletic group is the only clear instance of niche specialization in pneumococcus known to date. In this study, we compare the genomes and evolutionary histories of pneumococcal strains isolated from various body sites, focusing on multiple types of eye infections. We observe that conjunctivitis isolates cluster with a subset of nasopharyngeal isolates and away from all other strains, including those isolated from patients with keratitis and endophthalmitis. Phenotypically, our conjunctivitis isolates display aggregation in planktonic culture and chain-like structures in biofilms grown on an abiotic surface. Furthermore, we demonstrate that the *sspB* gene was acquired from *S. suis* by gene transfer and that its product plays a role in adhesion to the ocular epithelium. Our work combines comparative genomics, phylogenetics, and cell biology to explore the evolution and molecular mechanisms that underlie this unique instance of pneumococcus niche specialization.

The long branch that splits off the distinct phyletic group from other pneumococcal strains highlights the extensive differences in their core genomes and thus provides evidence of strain differentiation. This differentiation does not appear to be the consequence of niche separation, as some members of the distinct phyletic group are nasopharyngeal isolates and likely cocolonize with pneumococcal isolates from the main branch. Another mechanism promoting strain differentiation in pneumococcus and other bacteria is restriction-modification systems (52, 53). Whereas genomes in the distinct phyletic group do encode a type I restriction-modification system absent in the major branch, it is only encoded in a subset of these distinct strains (Fig. S1). This limited distribution suggests that this restriction-modification system is not driving strain differentiation. Thus, the molecular mechanisms driving this instance of strain differentiation remain a fascinating topic of study.

The strains in the distinct phyletic group share a set of genes not observed in strains outside this group. Do these differences in gene content between strains translate into differences in transmission routes and/or tissue tropism? Pneumococcal strains from the main branch are transmitted by nasal shedding, and the nasopharynx is the main reservoir of circulating strains (54). The phylogenetic grouping of conjunctivitis and nasopharyngeal strains is consistent with this model, in that strains from the distinct phyletic group may be transmitted to and from the nasopharynx and then disseminated to the ocular epithelium. Alternatively, strains acquired directly into the eye may have the ability to colonize the nasopharynx. Once in contact with the conjunctiva, a strain from the distinct phyletic branch may be able to overcome a specific host barrier and gain access to the ocular epithelium. Phylogenetic positioning of the keratitis and endophthalmitis isolates outside the distinct phyletic group suggests that this ability is not associated with other eye tissues or the ability to survive immunity in the eye. Instead, the ability to colonize the ocular epithelium could be guided by features specific to the conjunctiva, such as adhesion and/or colonization of its epithelium, or by an intermediate niche, such as eyelids, eyelashes, or even fingers that people use to rub their eyes. Moreover, these strains may be more resistant to desiccation or more competent at survival in the tear film, ultimately allowing productive infection of the conjunctiva. We postulate that the distinct phyletic group encodes proteins that allow these strains to overcome a host barrier associated with transmission and/or colonization of the ocular epithelium.

In this study, we captured a notable association between genotype and phenotype. First, we observed long chain-like structures only within biofilms of wild-type strains from this distinct phyletic group. To our knowledge, this phenotype has not been reported for biofilms of wild-type strains grown on abiotic surfaces. It has only been observed in cell culture (55) and with genetic mutants of *S. pneumoniae* (38, 56). Furthermore, there is precedence for a positive association between long chains and increased adherence (55), such that a similar trend may hold for strains in the distinct phyletic branch. Second, we observed aggregates in planktonic culture for the conjunctivitis strains. In a related experiment, Valentino and colleagues reported clumping of conjunctivitis isolates after addition of gp340, a glycoprotein found in tears (9). It remains to be tested whether these three phenotypes have the same molecular basis. Our studies with the *sspB* deletion demonstrate that SspB is not required for these phenotypes. These phenotypes could be due to other proteins, or perhaps the involvement of SspB is obscured due to redundancy in protein function.

Finally, our findings suggest that the gene encoding SspB contributes to adhesion to the ocular epithelium. It is likely that multiple redundant adhesins influence epithelial binding. For instance, a second putative adhesin with SspB-C2 domains (WP_050568636, previously referred to as SspB-C2 [9]) is also expressed in the distinct phyletic group (Fig. S1). The molecule contains multiple SspB-C2 domains (CDD E values of 1.89e−70, 1.8e−69, 2.17e−67, and 6.9e−66, respectively) and a C-terminal LPXTG motif, consistent with a cell wall attachment adhesin (Fig. S1). It seems probable that the propensity to form long chain-like structures in early biofilms and/or to aggregate in culture is widespread across isolates from the distinct phyletic group and, together with unique surface adhesins, plays an important role in interactions with the host.

The *sspB* gene is located within an ICE, and reconciliation between the streptococcal species tree and the *sspB* gene tree suggests *sspB* was acquired by gene transfer from *S. suis*. Furthermore, multiple *sspB* xenologs are contained within ICEs, such as MB56Spyo009, ICESde3396, ICEsu32457, and ICESsu32457 (57–60). Similarly, the reconciliation suggests these ICEs were also acquired by gene transfer from *S. suis*. Thus, it is likely that *S. suis* serves as an ICE reservoir dispersing these elements to many species within the streptococcal genus (60). It is tempting to speculate that ICE-associated adhesins may modify bacterial interactions with the host, either promoting or suppressing dissemination to specific hosts, body sites, and/or tissues.

The distinct phyletic group in the pneumococcal species tree provides a striking example of strain differentiation and tissue tropism. It generates many open questions on how these strains differ from other pneumococcal strains regarding evolution, transmission routes, morphology, gene expression, and host-pathogen interactions. Studies of these strains will provide exciting insight into the evolution and the biology of pneumococcus.

## MATERIALS AND METHODS

### Bacterial strains.

The *Streptococcus pneumoniae* isolates B1567, B1598, B1599, E709, K2521, K2527, and K2557 were obtained from patient ocular infections and stored by the Charles T. Campbell Eye Microbiology Laboratory at the University of Pittsburgh Medical Center (UPMC) Eye Center (Table 1).

### Bacterial growth conditions.

Frozen bacterial stocks were streaked onto Trypticase soy agar plates containing 5% sheep blood (BD BBL). All *S. pneumoniae* strains were grown in Columbia broth (Thermo Scientific) at 37°C with 5% CO_2_ without shaking. Medium was supplemented with antibiotics at 1 µg/ml for tetracycline and 100 µg/ml for spectinomycin.

### Pacific Biosciences SMRT sequencing.

Genomic DNA (10 µg) was extracted from strains E709, K2527, K2521, K2557, B1598, and B1599. Following digestion into ~10-kb fragments, the DNA was end repaired, purified with AMPure PB beads, and ligated to SMRTbell hairpin adapters. The SMRTbell libraries were further purified and quantified with a NanoDrop spectrophotometer and an Agilent 2100 Bioanalyzer. Polymerase-bound libraries were loaded onto a PacBio RS for sequencing with two SMRT cells per strain after completing primer and polymerase binding.

### Multilocus sequence typing of strains.

Sequences for the seven MLST alleles with available whole-genome sequences were extracted from sequence data and are listed in Table 1. For strain B1567, the ST was acquired by Sanger sequencing of PCR amplimers (21). For typing, the allele sequences were submitted to the *S. pneumoniae* MLST website (https://pubmlst.org/spneumoniae) (61). The serotypes were predicted by comparing the capsular locus to the sequences deposited in NCBI: specifically 15B (GenBank accession no. CR931664.1), 17F (GenBank accession no. CR931670), 19A (GenBank accession no. CR931675.1), and 23A (GenBank accession no. CR931683.1). Note that serotype 15C differs from 15B only by an additional two nucleotides within a stretch of TA repeats, wherein the additional bases appear to lead to a premature stop in a predicted *O*-acetyltransferase (62).

### Gene annotation.

Genomes were submitted to RAST for CDS prediction and annotation (63).

### Generation of *S. pneumoniae* species tree.

Forty streptococcal strains were selected for phylogenetic analysis (see Table S1 in Data Set S2). These genomes correspond to those used for the first large-scale pneumococcal pangenome studies (2, 5), as well as additional genomes from PCV-7-immunized children (22), and nonencapsulated strains (23). Combined these strains reflect a large variety of multilocus sequence types (MLSTs) and serotypes, as well as strains isolated from different disease states and geographic locations. The whole-genome sequences (WGSs) for all 40 strains were aligned using MAUVE (64), and the core region corresponding to 1,345,780 total sites and 92,728 informative sites was extracted from the MAUVE output files. Alignment of the core region was performed using MAFFT (MODEL) (65), and model selection was performed using ModelTest (66). The phylogenetic tree was built with PhyML 3.0, model GTR+I(0.63), using maximum likelihood analysis and 100 bootstrap replicates (67).

### Gene clustering and selection of genes unique to distinct phylogenetic clade.

The CDSs were organized into gene clusters as previously described (68). Briefly, similar genes were identified by tfasty36 (FASTA v.3.6 package) for six-frame translation homology searches of all predicted proteins against all possible translations (69). The output was parsed such that genes with at least 70% identity over 70% of their sequence were grouped into gene clusters allowing the strains to be analyzed for presence or absence of clusters. We selected genes present in strains B1598, B1599, MNZ14, MNZ85, and MNZ41 but absent in the remaining pneumococcal strains.

We utilized a set of 616 genomes isolated in Massachusetts from 2007 to 2010 (6) to compare our findings to previous work. This set, analyzed by Croucher and colleagues, contains 10 strains from a distinct phylogenetic branch termed SC12, as well as 606 additional genomes. To compare the sets, we used BLASTp and an E value cutoff of 1e−10 to query the 77 genes unique to our distinct phyletic group against a database of 1,231,516 sequences from the 616 genomes in the set isolated in Massachusetts from 2007 to 2010. The BLAST results were parsed to exclude hits with less than 70% identity and/or less than 70% coverage. A Python script was used to parse the BLAST output into a matrix correlating the query with the output. The data were represented as a heat map using the ggplot2 package in the R statistical package (70). All positive hits are plotted in Fig. S1. (Genomes without any hits are not represented in the heat map.)

### Phyletic distribution.

Sequences were retrieved using NCBI BLASTn to search the nonredundant (nr) database, restricted to *Bacillus*, *Lactobacillus*, and *Streptococcus* (taxid: 91061). The query corresponded to a 26,160-bp region of B1599 (within B1599_contig 202) that comprises *sspB* and adjacent genes. The resulting matches were curated to include only sequences with an E value of zero and a maximum bit score above 8,100; all of these matches entailed high-scoring pairs (HSPs) with at least 74% identity distributed along the full length of the query sequence, suggesting that these are genomic regions that are homologous to the query sequence in its entirety. The final set includes 26 genomic sequences, of which 5 are ICE sequences (see Table S3 in Data Set S2).

### Gene tree reconstruction.

To analyze the evolutionary origin of the *sspB* gene, we extracted the sequences that contain *sspB* and inferred a gene tree. Genomes lacking ORF predictions were discarded. For each of the remaining genomes, the *sspB* nucleotide and amino acid sequences were extracted from the GenBank file using GenBank_to_fasta.py, downloaded from the Rocab lab website (http://rocaplab.ocean.washington.edu/tools/genbank_to_fasta/). This resulted in 21 sequences after removing redundant sequences and adding B1599 *sspB* to the set.

Multiple alignment of the SspB protein sequences was performed using MAFFT (65), with the “E-INS-I” option via the Jalview dashboard (71). The alignment was converted into a codon-aware nucleic acid alignment using PAL2NAL (Fig. S1) (72), which was trimmed manually in Jalview to remove columns with more than 25% gaps. The best phylogenetic model for each of the three codon positions GTR + G, HKY + G, and HKY + G, was selected by the Bayesian information criterion using MODELGENERATOR (73) in Topali (74). A gene tree (Fig. 4A) was then constructed from the trimmed alignment using MrBayes v.3.2.6 (75); model parameters were fit by MrBayes for each codon site. The Markov chain Monte Carlo (MCMC) process was run for 500,000 generations with a sampling frequency of 15 generations and default settings for all other MCMC parameters. The gene tree was then midpoint rooted in FigTree, which was also used in figure generation (http://tree.bio.ed.ac.uk/software/figtree/).

### Gene tree-species tree reconciliation.

To infer the history of evolutionary events during *sspB* evolution, the resulting gene tree was reconciled with a species tree (Fig. S3), adapted from the tree from Richards et al. (50), for a reduced set of taxa. The Richards species tree was constructed from a concatenation of a core set of 136 genes across 44 streptococcus species (46 strains), representing 8 major groups. All species that encode a putative homolog of *sspB* were retained in the species tree for this study, as were additional species (that do not harbor a putative *sspB* homolog) to provide a broad representation of the streptococcal genus, including at least one species for each of the major taxonomic groups identified by Richards et al. (*mitis*, *sanguinis*, *anginosus*, *salivarius*, *downei*, *mutans*, *pyogenes*, and *bovis*). When an *sspB* homolog was predicted in more than one strain per species, all strains were added to the species tree. Strain relationships are unresolved in this tree; if more than two strains were included for a single species, their relationships are represented as a nonbinary node (i.e., polytomies).

We utilized Notung 2.9 to reconcile the *sspB* gene tree with this nonbinary species tree under a duplication, transfer, and loss model (see Fig. S5A in the supplemental material). Notung infers the history of events that minimizes the weighted sum of events when fitting a gene tree to a species tree; in this analysis, we used weights of 3.0, 1.5, and 1.6 for transfers, duplications, and losses, respectively. Notung does not infer events between taxa within an unresolved clade. We chose to represent strain relationships as nonbinary nodes (i.e., polytomies) in order to focus the analysis to interspecies transfers and not intraspecies transfers. A schematic (Fig. 4B; Fig. S5B to D) displaying the predicted history of gene transfers on the species tree was generated in FigTree and Adobe Illustrator.

### Biofilm growth and imaging.

All strains were grown in Columbia broth to an optical density at 600 nm (OD_600_) of 0.05 before seeding the culture onto MatTek dishes. At 24 and 48 h, medium was exchanged using diluted (1/5 with water) Columbia broth. At 72 h, biofilms were washed 2 times with phosphate-buffered saline (PBS) and fixed with 4% paraformaldehyde (PFA) for 30 min. Fixed biofilms were stained with Syto59 fluorescent dye according to the manufacturer’s instructions (LifeTech). Biofilms were imaged using a Zeiss 510 Meta Confocor3 laser scanning microscope, and images were processed using ImageJ.

### Bacterial cell aggregation assays.

Strains D39, B1599, and B1567 were inoculated into Columbia broth and grown until an OD_600_ of 0.05. Each culture was diluted 10-fold in full-strength Columbia broth and incubated overnight. Photographs were taken the following day, after 18 to 20 h of growth. Test tubes were photographed with Olympus Pen E-P1 for documentation.

### Construction of the deletion mutant and complemented strain.

The *sspB* deletion mutant in strain B1599 was generated by replacement of this gene with a spectinomycin resistance cassette. Specifically, we amplified the 1-kb upstream and downstream regions of *sspB* and ligated the flanking sequences to the resistance cassette by sticky end ligation with T4 DNA ligase. We amplified the ligation mixture by PCR to generate the transforming DNA (primers in Table S4 in Data Set S2). The *sspB* complement strain (B1599 Δ*sspB*::*sspB*) was generated in the Δ*sspB* background by reintroducing the *sspB* gene into a conserved intragenic region (contig 208, position 161851) previously used for complementation (53).

### Bacterial transformations.

For all bacterial transformations, about 1 µg of transforming DNA was added to the growing culture of a target strain at an OD_600_ of 0.05, supplemented with 125 µg/ml of CSP2 (sequence EMRISRIILDFLFLRKK [purchased from GenScript, Piscataway, NJ]), and incubated at 37°C. After 4 h, the treated cultures were plated on Columbia agar containing 100 µg/ml spectinomycin. Resistant colonies were cultured in media, the region of interest was amplified by PCR, and the amplicon was submitted for Sanger sequencing (Genewiz, Inc.) to verify the sequence of the mutants.

### mRNA isolation and qRT-PCR analysis.

Strains B1599, B1599 Δ*sspB*, and B1599 Δ*sspB*::*sspB* were grown in Columbia broth until reaching OD_600_s of 0.05, 0.2, and 0.5. At each time point, 5 ml of culture was collected and mixed with RNAlater. Pelleted cultures were frozen until RNA extraction. The RNeasy Plus minikit from Qiagen was used to extract and purify RNA from each sample. Each sample was DNase treated to remove DNA contamination. Expression of *sspB* was assayed by qRT-PCR and normalized to GAPDH (glyceraldehyde-3-phosphate dehydrogenase). Primers for each locus were designed using Roche Universal Probe Library software. The experiment was performed in triplicate, and data were analyzed using LinReg PCR software. Statistical analysis was performed with Wilcoxon’s paired rank test using GraphPad.

### Mammalian tissue culture conditions.

Human corneal limbal epithelial (HCLE) cells were cultured in keratinocyte serum-free medium (KSFM [Gibco catalog no. 10724-011]) containing 25 μg/ml bovine pituitary extract (Gibco catalog no. 13028-014), 10.2 ng/ml embryonic growth factor (Gibco catalog no. 10450-013), 100 µg/ml penicillin, and 100 µg/ml streptomycin (Corning catalog no. 30-002-CL).

### Bacterial attachment to HCLE cells.

HCLEs were seeded into 12-well MatTek glass bottom dishes (MatTek P12G-1.5-14-F) in antibiotic-free KSFM at a density of 1.50 × 10^5^ cells per well and allowed to adhere overnight at 37°C with 5% CO_2_. *Streptococcus pneumoniae* strains B1599 (wild type), B1599 Δ*sspB*, and B1599 Δ*sspB*::*sspB* were streaked onto blood agar and grown overnight at 37°C with 5% CO_2_. Bacteria were scraped off of the blood agar with an inoculating loop, added to 2.5 ml Columbia broth, and grown for 5 h to an OD_600_ of 0.3. Cultures were pelleted by centrifugation at 14,000 rpm for 2 min. The pellets were washed two times in PBS and resuspended in 1 ml Columbia broth. Two hundred microliters of each strain was added to each well of HCLE cells containing 1 ml of antibiotic-free KSFM. The plate was incubated at 37°C with 5% CO_2_ for 30 min. After incubation, HCLEs were washed gently two times with PBS and supplemented with fresh KSFM. The cells were imaged on an Olympus Fluoview FV-1000 laser scanner confocal microscope with a 60× objective. Ten fields per treatment group were imaged, and the number of bacteria on each HCLE was manually counted using Fluoview image viewing software version 3.1. The experiment was repeated on three separate days with similar results.

### Accession number(s).

We have submitted the complete genome sequences of the following six genomes to GenBank under the accession numbers given in parentheses: E709 (JBOR00000000), K2521 (JBOS00000000), K2527 (JBOT00000000), K2557 (JBOU00000000), B1598 (JBOV00000000), and B1599 (JBOW00000000).
