# Supplementary figures and images for: Gene Acquisition by a Distinct Phyletic Group within Streptococcus pneumoniae Promotes Adhesion to the Ocular Epithelium
Source: mSphere. 2017 Oct 25;2(5):e00213-17. doi: 10.1128/mSphere.00213-17 (PMC5656748; doi:10.1128/mSphere.00213-17)

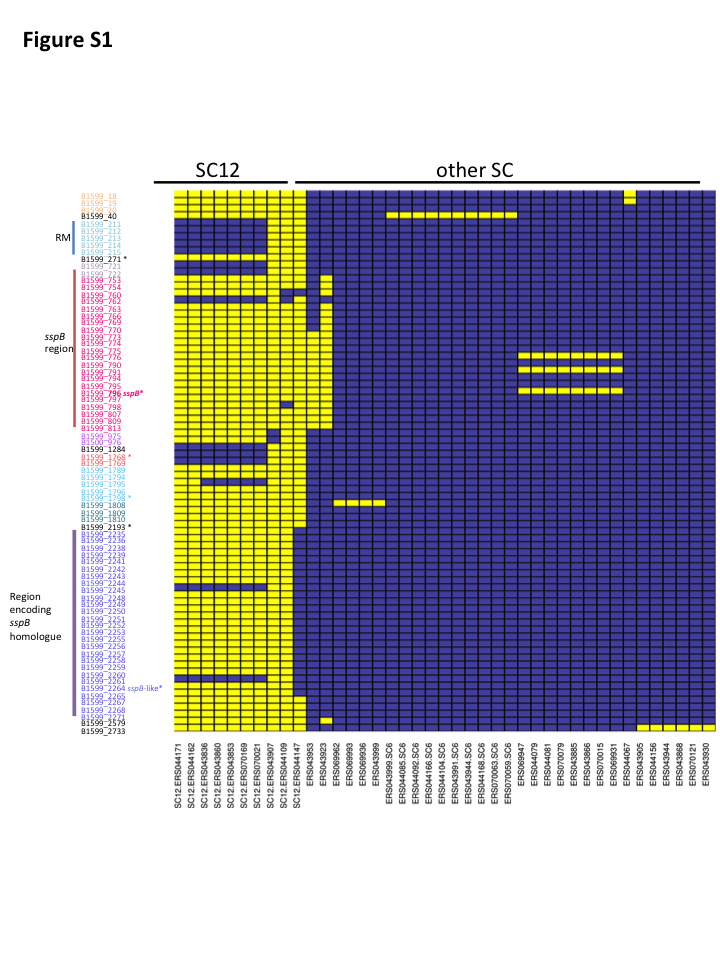

Supplement: FIG S1 [file sph005172385sf1.tif]

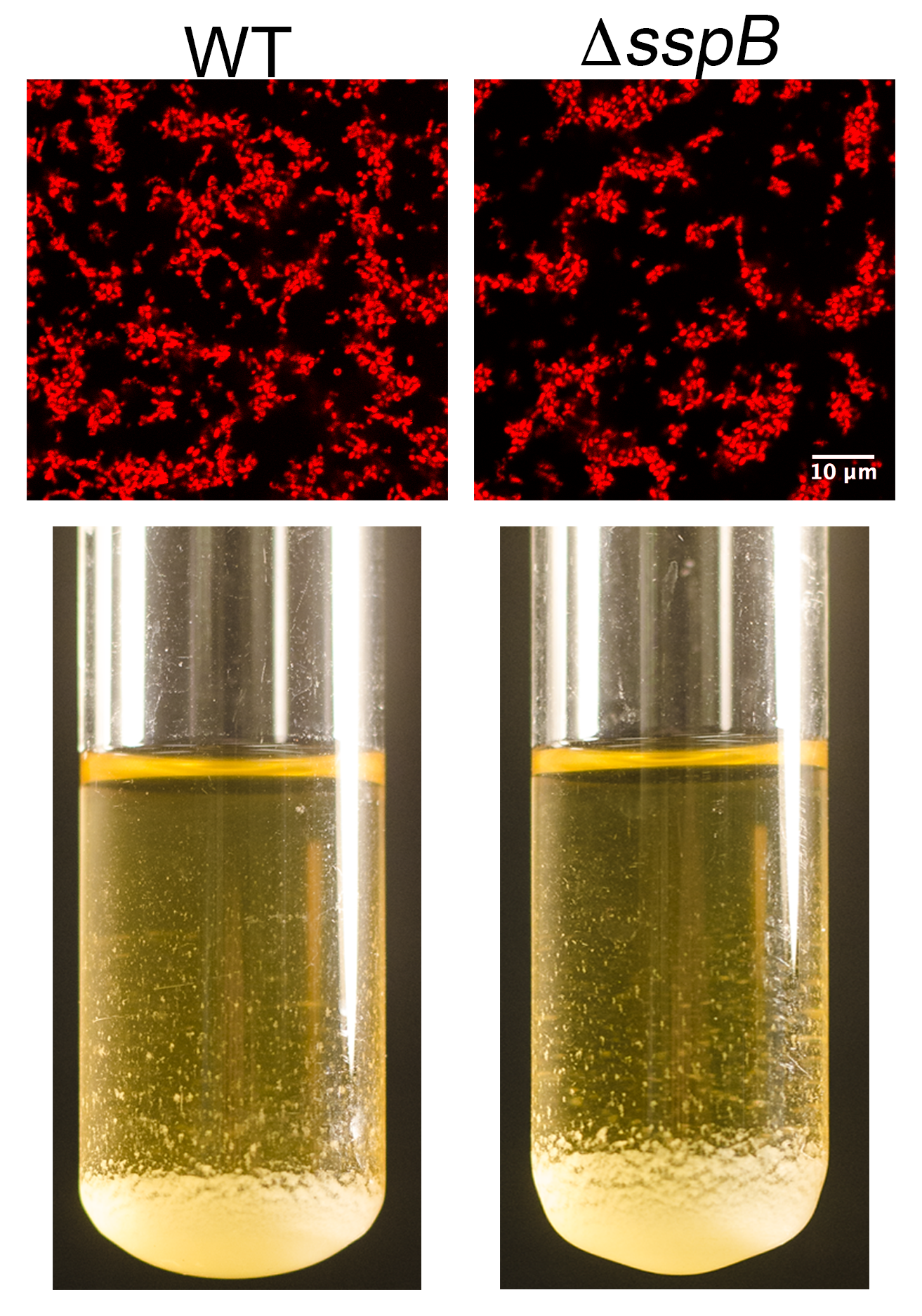

Supplement: FIG S2 [file sph005172385sf2.tif]

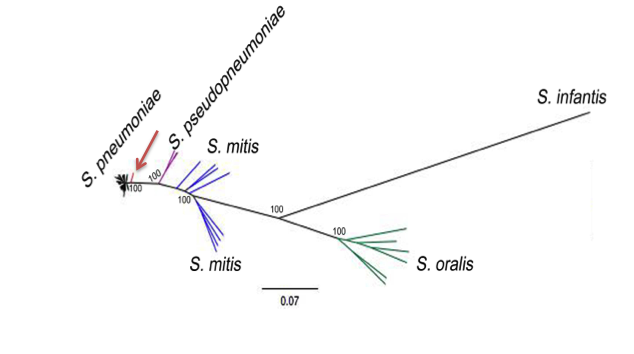

Supplement: FIG S3 [file sph005172385sf3.tiff]

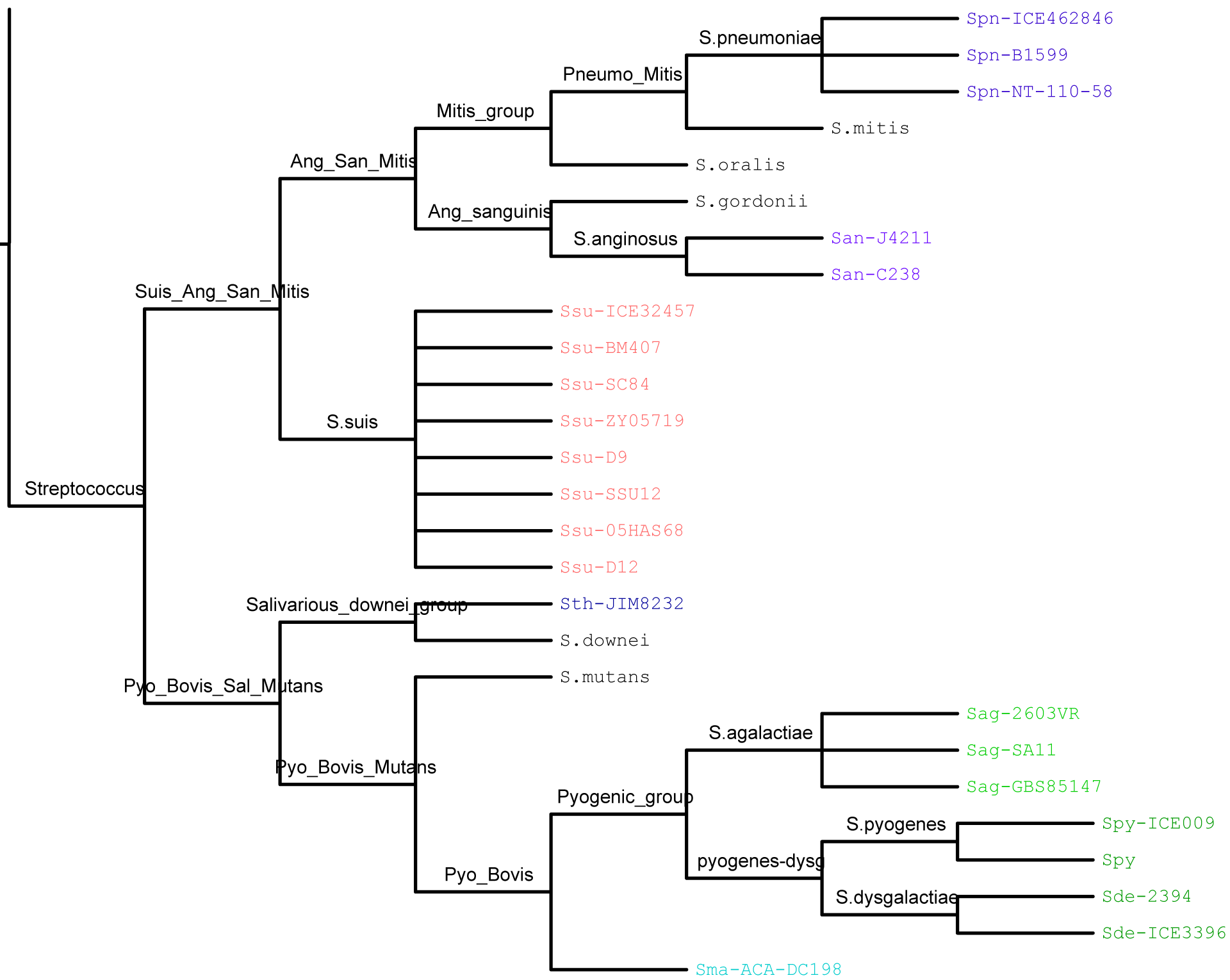

Supplement: FIG S4 [file sph005172385sf4.tif]

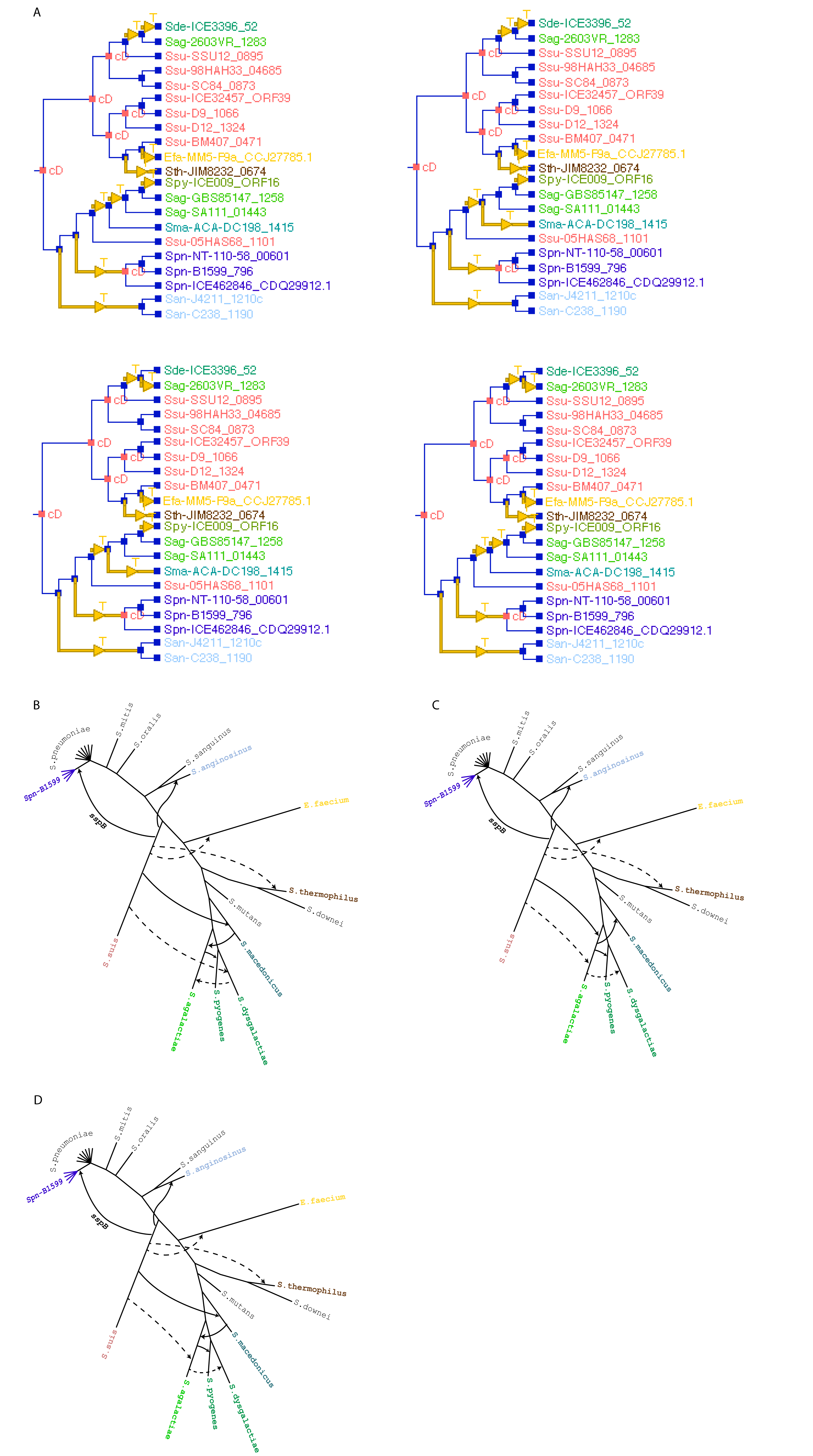

Supplement: FIG S5 [file sph005172385sf5.tiff]
